# Medical student perceptions and experiences of incivility: a qualitative study

**DOI:** 10.1186/s12909-023-04354-6

**Published:** 2023-06-05

**Authors:** Louise Griffin, Anna Baverstock

**Affiliations:** 1 Birmingham Medical School, University of Birmingham, Birmingham, UK; 2 Musgrove Park Hospital, Somerset Foundation NHS Trust, Taunton, TA1 5DA UK

**Keywords:** Incivility, Bullying, Discrimination, Medical students, Medical education, Qualitative methods, Mental health

## Abstract

**Background:**

Incivility is rude, dismissive or aggressive behaviour in the workplace. Rates of incivility are increasing in healthcare settings, with minority groups at greatest risk. Medical students are particularly vulnerable to incivility whilst on clinical placements, with detrimental consequences on students’ learning and mental health. Therefore, this study explored the perceptions and experiences of incivility from healthcare workers amongst medical students.

**Methods:**

An online qualitative questionnaire study employing a thematic analysis. Students in years 3–5 or intercalating at a large West Midlands medical school were recruited between May–June 2022. Qualitative free-text questions explored students’ experiences of incivility from healthcare workers over the past 12 months, and actions in response to incivility. Data were analysed using thematic analysis. All researchers agreed thematic saturation was reached at 50 responses, with all year-groups represented.

**Results:**

Five core themes were identified: abuse of *hierarchy; exclusion; discrimination; response to incivility; barriers to action.* Participants reported a range of uncivil behaviour from staff, including mocking, exclusion and discrimination. Discriminatory incivility targeted students’ protected characteristics, including race, sex, sexual orientation and disability. In response to experiencing or witnessing incivility, participants varied in their preferred mode of action. Many viewed formal escalation to senior staff as favourable action. Meanwhile, other participants would not escalate concerns and instead respond with peer support or allyship. Marked barriers prevented students from challenging or reporting incivility, including a lack of confidence; failures and fears of reporting systems; and perceived severity of abuse.

**Conclusion:**

Our findings identify the extensive incivility experienced by medical students on clinical placements, which is frequently perpetuated by abusive workplace hierarchy. Whilst students recognise the importance of reporting uncivil behaviours, barriers to reporting include unrecognised incivility, psychological consequences and failed reporting systems. In order to reform toxic educational environments, we recommend medical schools to integrate formal civility training into the curriculum and restructure accessible, supportive reporting systems.

## Background

Incivility in the workplace was first conceptualised by Andersson and Pearson [1] and has since been widely documented within the healthcare setting [2]. Incivility is broadly recognised to represent a rude, aggressive or dismissive behaviour of ambiguous interpretation, which violates workplace expectations [1, 3]. Examples of uncivil behaviour include: public humiliation; condescending language; belittling; ignoring others [4]. Discrimination on the basis of a protected characteristic also falls under the umbrella of incivility, termed as ‘selective incivility’ [5].

In recent years, growing rates of incivility have been reported by healthcare workers (HCWs), as demonstrated by 18.7% of NHS staff experiencing harassment, abuse or bullying by a colleague in 2021 [6]. Such incidents are rarely isolated and can occur multiple times per week [7]. Certain minority groups are at increased risk, with a greater proportion of disabled (+ 8.7%) and minority ethnic staff (+ 5.1%) reporting incivility compared to their respective non-disabled and White colleagues [6]. A recent British Medical Association report revealed widespread racism experienced by 76% of doctors, leading many to consider leaving the profession altogether [8].

The impact of incivility has been widely recognised to jeopardise clinical performance, patient safety and staff well-being. Exposure to uncivil behaviour impairs the performance of both recipients [9] and onlookers, [10] culminating in deficient team collaboration [11]. This, in turn, risks patient safety and is repeatedly linked to adverse ‘iatrogenic’ events, such as medical errors and mortalities [12, 13]. Moreover, repeated subjection to incivility places HCWs at risk of psychological distress, [7] compassion fatigue and subsequent burnout [14].

Medical students are particularly vulnerable to experiencing incivility from other HCWs whilst on clinical placements. This is largely due to frequent rotation changes and working with unfamiliar staff. Healthcare hierarchy further exposes students to greater rates of uncivil behaviour compared to their seniors, who remain relatively protected by status [7, 15]. Studies consistently point to high rates of incivility experienced or witnessed by medical students, [16–19] which are commonly conducted by senior perpetuators [20]. These incidents remain largely unreported by students, due to a fear of repercussions in a hierarchal learning environment [17]. As well as inhibiting clinical learning, [21] incivility can lead to psychological distress and burnout [22, 23]. Existing evidence reveals a hidden curriculum which socialises medical students to expect and potentially model uncivil behaviour, [17] risking a continuation of uncivil workplace culture and its subsequent negative impact.

To date, there is limited discussion surrounding the perceptions and qualitative experiences of incivility within the UK medical student population. Yet, the detrimental impact of incivility on students’ learning and mental health highlights the need for further research. Identifying the nature of incivility experienced by students is essential in developing greater civility awareness and accessible reporting systems. Therefore, this study will explore the experiences of incivility from HCWs amongst UK medical students.

Our aims are to:

1. Explore the experiences of incivility from staff amongst medical students on clinical placements.
2. Explore the actions and reporting behaviour of medical students following uncivil behaviour.
3. Inform recommendations for civility awareness in medical education and student reporting systems.

## Methods

### Study design and setting

An online qualitative questionnaire study design employing a thematic analysis to facilitate an in-depth exploration of the perceptions and experiences of incivility amongst medical students. The study setting was a large medical school in the West Midlands, providing undergraduate, graduate and intercalation programmes. Years 3–5 constitute the ‘clinical’ years, during which education is delivered in hospital settings.

### Questionnaire development

Many existing incivility assessment tools are quantitative in nature and not suited to explore the individual experiences and subsequent actions of victims [24]. Consequently, a qualitative questionnaire was devised, with its questions relating to participants’ experiences and actions. This was informed by incivility literature and the expertise of AB who has prior research experience in this area [17, 18, 25, 26]. This was piloted amongst medical students to ensure questions were suitable and comprehensive. Modifications were made based on pilot feedback. The final questionnaire contained qualitative free-text questions, which explored participants’ experiences of incivility over the past 12 months and their actions in response to incivility.

### Recruitment

Students in years 3–5 (including those intercalating) were recruited via social media, email bulletins and student-led societies. Study information was provided and informed consent gained from all participants. Participants were assured of their confidentiality and that they would not be identifiable from data provided. The MBChB University of Birmingham Research Ethics Committee approved the exemption for ethical approval and provided guidance throughout the research process.

### Data collection

The online questionnaire, hosted on Microsoft Forms, was distributed amongst eligible students between May–June 2022. A total of 50 participants completed the questionnaire, at which point all researchers agreed that thematic saturation had been reached and recruitment was stopped [27].

### Data analysis

Questionnaire data was imported into Microsoft Excel and stored on a secure server. Data were analysed using the thematic analysis method of Braun and Clarke with an inductive approach [28]. This analysis occurred simultaneously alongside data collection, ensuring iterative practice until data saturation was achieved and no new data were generated [28]. LG analysed all questionnaire responses independently, creating preliminary codes according to content. Codes were repeatedly developed and refined following analysis of new data. Alongside regular researcher discussions, AB independently reviewed the coded questionnaire responses. LG employed constant comparison [29] to identify preliminary themes according to observed data patterns. Both researchers further refined and agreed upon the final descriptive themes.

### Participant wellbeing

It was recognised that the sensitive subject matter and disclosures of incivility may raise concerns amongst participants. Therefore, information sign-posting students to wellbeing services was clearly provided during study advertisement and on questionnaire completion.

## Results

A total of 50 medical students completed the questionnaire between May–June 2022. The year-groups represented by participants are described in Table 1. Accounts of incivility in the past 12 months were reported by 84% of participants (*n* = 42).

**Table 1:** Demographics of participants

Participant year-group	*N* = 50 [% frequency]
Year 3	10 [20]
Year 4	21 [42]
Year 5	12 [24]
Intercalating between Year 3/4	2 [4]
Intercalating between Year 4/5	5 [10]

Thematic analysis of qualitative responses, relating to participants’ experiences over the past 12 months, identified 5 main themes: abuse of *hierarchy; exclusion; discrimination; response to incivility; barriers to action.* These are described below with accompanying quotes.

### Abuse of hierarchy

Twenty-two students reported incivility as a result of healthcare hierarchy, which facilitated staff to assert their seniority in an abusive manner, as this participant indicates:

*“I was told to leave an assessment unit as I would ‘slow the doctors down’ and would get in the way, this was said from a senior doctor to a junior doctor that was happy for me to shadow and support, it wasn’t even said to me directly”* (P7, fifth-year).

Incivility from senior HCWs acting within this hierarchy frequently involved remarks belittling students’ competencies:

*“[I] have been called stupid, laughed at, had the doctor told to not let me do any skills because I'm clearly incompetent”* (P27, fourth-year).

The mocking of students and other staff was another form of incivility described by participants. These accounts often involved a public display of humiliation, with an intended wider audience of patients and/or staff:

*“I remember a surgeon telling me I wasn't going to be a good doctor in front of my firm [other students] and the patient after I forgot to ask a few questions in the history”* (P13, third-year).

Again, such behaviour was associated with undermining students’ capabilities and confidence.

### Exclusion

Alongside active uncivil behaviour, 12 participants described experiencing incivility in the form of exclusion or dismissal. When attending clinical environments, often for important and prescribed learning opportunities, students were met with rejecting behaviour:

*“[I] walked onto a ward, expecting to be helped, very politely asked a doctor to help us - she didn’t even look at us and said 'go ask the other doctor', she was very very unwelcoming, very hostile, very rude and was very discouraging”* (P24, third-year).

Some students were completely ignored altogether, as this participant indicates:

*“doctors and nurses seem to ignore me when I ask for help or to help with tasks”* (P19, third-year).

### Discrimination

The most frequently reported incivility was of a discriminatory nature, related to protected characteristics including sex, race, age, religion and disability. Fifteen students described accounts of racism, sexism, homophobia or ableism by other HCWs:

*“medical student implied that an international medical student should go back to where they come from and not train here”* (P4, third-year).

*“a consultant ‘joking’ about how women would be good at curling in the Olympics because they are good at sweeping around the home”* (P30, fifth-year).

Ableist behaviour involved objectifying participants as ‘practice’ patients with one participant described as *“the damaged goods”* (P11, fourth-year) by a teaching doctor. This discrimination often demeaned students by calling into question their abilities to become doctors:

“…*nurses and doctors have told me I should consider another career. One doctor told me patients don't want patients looking after them.”* (P49, fourth-year).

One participant highlighted that their lived experience of selective incivility was conceived of overlapping protected characteristics:

*“The intersectionality of disability, race and religion can make placement a place of perpetual bullying. I'm always on edge.”* (P48, fourth-year).

### Response to incivility

Following an incident of incivility, 50% of participants stated a lack of confidence to act. Only 3 students reported uncivil behaviour they had witnessed or experienced to senior leadership. Meanwhile, when asked about how they would respond to future incivility, 26 participants cited escalation to senior staff as most suitable:

*“Ideally, should report the behaviour or seek help to address it”* (P39, intercalating between years 3/4).

Eighteen participants indicated a desire to respond with peer support or allyship, as opposed to escalating action. Following an uncivil event, providing or seeking peer support was the preferred first step before considering alternative action. However, some students stated they would not continue to escalate concerns after this:

*“I would try to speak to friends and talk about how I feel. I don't think I would report it to the medical school.”* (P19, third-year).

Other participants described different forms of allyship, including directly challenging uncivil behaviour *“in a polite way”* (P32, fifth-year), removing *“the person affected from the situation”* (P4, third-year) or supporting victims to *“report to the undergrad[uate] department”* (P42, fourth-year).

### Barriers to action

Although many participants described active responses to incivility, it is important to highlight that nearly 50% of students cited barriers to challenging or reporting incivility. Emotional exhaustion prevents students from reporting abuse, particularly following repeated exposure:

*“My personal experiences have depleted my resilience and emotional strength. I doubt I would be able to pursue action in defence of myself.”* (P11, fourth-year).

This has resulted in some participants accepting incivility as the status quo of medical education:

*“At this point, I'm so worn down. I'd probably accept it as part of placement and move on.”* (P48, fourth-year).

Other barriers to formally reporting incivility included perceived negative repercussions and failures of existing support systems. One participant stated their response to incivility would be to:

*“cry because what else can you do when a 60 year old doctor is belittling you, you get in trouble for speaking out”* (P27, fourth-year).

Meanwhile, others described a lack of action from educational bodies (such as hospital or university staff), particularly if behaviour is perceived as ‘minor’ aggression:

*“[it] does not feel like they will help you much unless it is major”* (P31, fifth-year).

Consequently, this deters students from raising concerns.

The context surrounding an uncivil incident was repeatedly highlighted as the main determinant of an individual’s response. Eight participants dictated action by a sliding scale of behaviour severity, with self-identified ‘major’ incidents warranting escalation:

*“Depends on how bad it is, if I am ignored for example, I am less likely to take action. But if I witness incivility towards other people especially based on protected characteristics, I would definitely report that and try to support the person.”* (P44, fourth-year).

The victim’s identity provided a different context for other participants who felt empowered to support peers but not themselves:

*“If it affected a friend I would support them in to follow through medical school guidance. For myself, I have little faith in the system and know this is part of medicine.”* (P49, fourth-year).

## Discussion

### Experiences of incivility

These findings identify incivility as a common experience amongst UK medical students on clinical placements, including mocking, humiliation, dismissal and discrimination. This is consistent with the growing body of evidence reporting high rates of incivility within medical student populations [19, 30–32].

We propose medical students on clinical placements possess specific vulnerability to incivility, additional to that of other HCWs. Existing data reports alarmingly high mistreatment of students, [19, 30–32] which is noted to underrepresent true figures [19]. This greater risk is likely due to a culmination of factors unique to the student identity. The power imbalance dictating teacher-student encounters provides a breeding ground for incivility to occur and remain unchallenged. Furthermore, frequent placement rotations see students working in new and unfamiliar environments, without the ability to ever fully integrate into the healthcare team. Again, this leaves students vulnerable to experiencing incivility and without the tools to seek support or escalate concerns.

The abuse of hierarchy forms a common thread between participants’ experiences of incivility. Status disparity exposes students as convenient targets of incivility, whilst simultaneously disarming victims from challenging behaviour or seeking support. Rates of incivility reported by medical students are comparable to those experienced by junior doctors [17, 33]. Given both groups share similar ‘junior’ identities in the workplace, this further suggests that hierarchy drives incivility. Indeed, a hidden curriculum of hierarchy acceptance and subsequent emotional numbing [34] has been identified as a key source of incivility and underreporting in medical education [19]. Acts of incivility create a way by which hierarchy can be learnt and reinforced [34].

Experiences of selective incivility targeting students’ protected characteristics comprised the majority of reported incivility. Participants’ accounts of discrimination spanned racism, sexism, ableism and homophobia. This echoes the extensive documentation of discrimination as a widespread phenomenon in medical education [18, 35–38]. Our findings of racism are particularly timely given recent reports of racial harassment within UK medical schools [8, 39]. Early and repeated exposure to discrimination could push students to consider alternative career pathways and leaving the profession.

It is essential to consider these experiences of incivility within an intersectionality framework, as first theorised by Black feminist Kimberlé Crenshaw, [40] in order to fully illuminate the structural processes driving uncivil behaviour and victims’ experiences [41]. Despite one participant referencing intersectionality, our findings fail to account for intersectionality and we cannot comment on its influence on participants’ marginalisation. Future research must move away from reporting discrimination within an isolated context, but instead consider the intersectionality of incivility by exploring the layering of experiences.

### Impact of incivility

Our findings of extensive incivility experienced by medical students are of concern when considering the impact on victims’ psychological wellbeing. Abusive behaviour is recognised to negatively impact on students’ self-esteem and confidence surrounding their professional identity [16]. Adverse psychological consequences, including anxiety, low mood, alcoholism and suicidal ideation, have also been linked to severe harassment [42, 43]. Repeated exposure to incivility is likely to contribute to student burnout, owing to its subsequent emotional fatigue and depersonalisation [23]. This link between incivility and burnout has growing relevance amidst rates of student burnout consistently rising above 50% [44, 45]. Incivility may play a more sinister role in students’ exhaustion and subsequent early departure from the medical profession.

It is essential to highlight the pervasive nature of incivility at the undergraduate level, with 84% of participants experiencing or witnessing incivility. Alongside previously high reports of incivility, [31, 32] our findings speak of a harmful culture within healthcare education which socialises students to accept, expect and practise incivility. In order to survive the hostile workplace, the abusive attitudes and behaviours of superiors are adopted by students [46]. The student experience of incivility cycles for generations, whereby victims become perpetrators [47]. Additionally, students are taught a complementing curriculum of silence, self-sacrifice and toxic resilience in the face of mistreatment [19]. Despite growing awareness of problematic medical culture and counter-efforts by educational bodies, [48] a required intergenerational shift is yet to break this cycle.

### Barriers to reporting

In response to experiencing or witnessing incivility, participants most commonly cited reporting the incident to senior staff as their preferred mode of action. However, 50% of participants described a lack of confidence preventing speaking up and action. Peer support, active bystander and allyship were highlighted as alternatives due to the barriers of formal escalation. This is unsurprising given substantial evidence demonstrating peers as the most common source of support sought by students [49].

The underreporting of incivility within healthcare is a potentially devastating phenomenon which disproportionately affects medical students and minority groups [50]. A 2018 report found only 43% of students felt confident reporting incidents of incivility [50]. The barriers to reporting described by participants help to explain this gulf between reported and experienced incidents. The challenges of reporting incivility stem back to its ambiguous nature, [1] the subtilties of which further undermine and disempower victims. This may explain participants’ perceptions that mistreatment was not severe enough to warrant support or escalation. Incivility is defined by how something is heard or felt and therefore by its nature can be challenging to recognise and report.

The psychological impact of incivility, as previously discussed, facilitates an acceptance of uncivil behaviour as the status quo. The draining emotional consequences leave little room for victims to report their experiences at a time of vulnerability. This highlights a failure of existing reporting systems, which place onus on victims to initiate action. Reporting is widely perceived as futile amongst medical students [42]; a perception shared by our participants due to previous negative experiences. For those that do consider formal avenues of reporting, the fear of adverse repercussions prevents students from raising concerns, [51] including: antagonising further incivility; negatively affected grades; or prolonged suffering [20]. Together, these factors cultivate a culture of silence deterring students from speaking out.

### Recommendations

The extensive nature of incivility and its potential detrimental impact on medical students highlights the urgent need for improved incivility awareness, reporting systems and wellbeing services. As the educational bodies responsible for medical student welfare, medical schools and teaching hospitals must formally acknowledge the scale of incivility experienced by their students.

Reforming current toxic culture requires in part a top-down approach, [52] whereby educational leaders cultivate respectful learning environments and safer forums to raise concerns. We also need to create brave spaces where these concerns can be discussed. We recommend the integration of civility training into the medical curriculum to improve civility awareness amongst students. Such training should: (i) define incivility; (ii) explore the impact on victims, staff and patients; (ii) discuss ways to safely respond to incivility; (iv) sign-post to formal support. Previous implementation of similar programmes has been successful in increasing civility awareness and attendees’ confidence to respond to incivility [53, 54].

Additionally, medical schools must reshape current systems for reporting and processing complaints. Students need to have the options to remain anonymous and be actively involved in the response proceedings [48]. Systems could incorporate planned, regular feedback on clinicians at the end of placement rotations to remove the barriers and negative connotations of reporting [55]. Furthermore, reporting infrastructure must work closely with wellbeing services to ensure support remains visible and accessible for victims.

Future research should investigate the experiences of incivility and its impact on medical students at a national level, utilising qualitative focus groups or interviews to explore the subject’s complexities.

### Limitations

Our study has several limitations. Firstly, the study sample is small and selected from a single UK medical school setting, limiting the transferability of our findings. We devised our own qualitative questionnaire, which has not been trialled or validated by external parties. Students with experiences of incivility may have been more likely to participate, which may have skewed our data towards negative reporting. Contrastingly, participants were required to self-identify experiences as incivility, which may have underrepresented students’ mistreatment. Finally, whilst the anonymity and freedom of open questions provided a safe forum to share sensitive responses, this methodology prevented the ability to probe and ascertain richer data. Consequently, we were unable to consider the broader context of incivility, including situational data related to speciality or environment.

## Conclusion

Our results suggest medical students experience high levels of incivility from HCWs whilst on clinical placements, including mocking, exclusion and discrimination. Healthcare hierarchy drives both this incivility and its underreporting. Whilst students recognise the importance of reporting uncivil behaviours, several barriers deter students from formally escalating concerns and to instead seek peer support. Misconceptions of incivility, associated psychological challenges and failures of reporting systems all contribute to the underreporting of incivility. The detrimental impact of incivility on students’ psychological wellbeing and the subsequent reinforcement of toxic workplace culture highlight the need for systemic change alongside the creation of brave spaces for learning described above. Medical schools and teaching hospitals are encouraged to lead a cultural shift towards respect, compassion and safety for all students. This can be achieved through the integration of formal civility training into the medical curriculum and restructuring of reporting systems to reduce access barriers and support victims.

## Data Availability

The datasets generated during the current study are not publicly available due to the personal nature of the topic, but are available from corresponding author on reasonable request.

## Abbreviation

HCW: Healthcare worker
